# Drinking Among Young Adults: Prevalence, Patterns, and Consequences

**Published:** 1996

**Authors:** Lori A. Quigley, G. Alan Marlatt

**Affiliations:** Lori A. Quigley, Ph.D., is a postdoctoral research associate trainee at the Addictive Behaviors Research Center and G. Alan Marlatt, Ph.D., is a psychology professor and director of the Addictive Behaviors Research Center, Department of Psychology, University of Washington, Seattle, Washington

**Keywords:** AOD use pattern, AOD consumption, AOD associated consequences, risk factors, young adult, prevalence, college student, binge AOD use, heavy AOD use, AOD abuse, AOD dependence, drinking and driving, literature review

## Abstract

Young adults have a higher prevalence of alcohol consumption and binge drinking than any other age group. They also drink more heavily and experience more negative consequences of drinking. Rates of alcohol abuse and dependence are disproportionately higher among those between the ages of 18 and 29 compared with other age groups. Young adults are also overrepresented among alcohol-related traffic fatalities. Over time, distinct patterns of change in frequent binge drinking occur, and most heavy-drinking young adults appear to “mature out” of abusive drinking patterns as the responsibilities of later adulthood supervene. Drinking patterns are affected by demographic, psychological, behavioral, and social factors as well as minimum drinking age legislation and the cost of alcohol. Motivational programs designed to reduce risks and consequences associated with young-adult drinking may help in reducing alcohol consumption and its consequences.

Young adults have a higher prevalence^1^ of alcohol consumption than any other age group. They also drink more heavily, experience more negative consequences of drinking, and engage in more activities while drinking that may put them at risk for many types of harm. Surveys have documented a decline in alcohol consumption among all age groups in recent years, including young adults (Johnston et al. 1996; Midanik and Clark 1994; Substance Abuse and Mental Health Services Administration [SAMHSA] 1994; Williams and Debakey 1992). However, consumption rates remain highest from the late teen years to the late twenties (Johnston et al. 1996; SAMHSA 1994).

This article summarizes results of several studies regarding the prevalence, patterns, and consequences of youthful drinking and provides an overview of risk factors for problem drinking. Many studies of young adults use samples composed exclusively of college students. Therefore, where possible, this review draws from studies that are national in scope and not focused predominantly on college populations.

## Extent of Alcohol Consumption

Table 1 presents findings derived from two national surveys. One, Monitoring the Future (Johnston et al. 1996), focuses on drug use by young adults. Among high school graduates ages 19 to 28, a total of 91.2 percent reported consuming alcohol at some time in their lives (i.e., lifetime prevalence). Within this same group, 83.7 percent reported alcohol consumption in the past year, and 67.7 percent had consumed alcohol within the past 30 days.

The second survey, the National Household Survey on Drug Abuse (NHSDA) (SAMHSA 1994), found similar results. Participants ages 26 to 34 reported higher lifetime, annual, and past-month prevalence of alcohol use than did young adults ages 18 to 25. Lifetime, annual, and past-month alcohol use among young adults surveyed were dramatically higher than among respondents ages 12 to 17. Annual and past-month prevalence rates drop off precipitously after age 35, indicating that the highest recent prevalence of drinking is among those who are in the young-adult age range.

## Extent of Binge Drinking

Binge drinking, or discrete episodes of heavy drinking, is common among youthful populations. The rate for binge drinking, defined by the Monitoring the Future study as consumption of five or more drinks on one occasion at least once within the 2-week period preceding the survey, was 34.4 percent 1 to 4 years after graduation from high school. The highest rate, 41 percent, was among 21- to 22-year-olds (Johnston et al. 1996).

Young adults in college have a higher rate of binge drinking (40 percent) relative to their noncollege peers. This difference may reflect easier access to alcohol and parties among students, as well as non-college students’ earlier adoption of adult roles involving work or marriage. Relative to high school seniors, these rates reflect a 20-percent increase in binge drinking for young adults not in college compared with a 42-percent increase for young adults in college (Johnston et al. 1996). This trend toward heavy drinking appears to begin before college, however. In fact, those who are among the heaviest drinkers in high school are likely to continue drinking heavily following graduation, whether or not they plan to enter college (Baer et al. 1995). Surprisingly, college-bound high school seniors report binge drinking in high school *less* frequently than non-college-bound students (Johnston et al. 1996).

In the NHSDA, 18- to 25-year-old respondents showed a lower prevalence of alcohol consumption than the 26- to 34-year-old cohort. With regard to patterns of heavier consumption, however, the younger group reported a higher prevalence of heavy drinking or frequent monthly binge drinking. The rates of frequent monthly binge drinking, defined as drinking five or more drinks per day on each of 5 or more days in the past 30 days, are presented in table 1 (SAMHSA 1994). According to the NHSDA, heavy drinking increases during young adulthood and declines somewhat after the mid-thirties.

Gender differences are also apparent in binge-drinking rates (Johnston et al. 1996; SAMHSA 1994). Men between the ages of 19 and 32 were more likely to be binge drinkers than were women, based on a binge-drinking criterion of five or more drinks on one occasion in the previous 2 weeks (Johnston et al. 1996). The five-drink criterion, however, has been shown to underestimate the prevalence of binge-drinking among women (Wechsler et al. 1995*a*). A four-drink binge criterion for women has been recommended by Wechsler to correct for gender differences in body mass and alcohol metabolism and to approximate equal likelihood of drinking-related consequences between genders (Wechsler et al. 1995*a*). The application of this criterion would result in a binge-drinking rate of 39 percent among young-adult college women, which is considerably higher than the 33-percent rate that would result from the five-drink criterion (Wechsler et al. 1995*a*).

Ethnic and regional differences in frequent monthly binge-drinking rates also have been documented. White NHSDA respondents most often reported frequent monthly binge drinking, followed by Hispanic and African-American respondents (SAMHSA 1994). A pattern of frequent binge drinking was reported less often by respondents living in the southern region of the United States compared with those in the northeastern, north central, and western regions (SAMHSA 1994). Similarly, Wechsler and colleagues (1994) found that colleges in the northeast and north central regions had higher rates of binge drinking than those in the South and West.

## Consequences of Drinking Among Young Adults

### Alcohol Abuse and Alcohol Dependence

Not only do young adults have a higher prevalence of alcohol consumption and binge drinking than other age groups, they also are overrepresented in diagnosable rates of alcohol-related disorders (i.e., alcohol abuse and alcohol dependence). The criteria for these disorders reflect in part the physical, occupational, interpersonal, and psychological consequences associated with the use of alcohol. Criteria for diagnosing alcohol abuse and alcohol dependence are outlined in the *Diagnostic and Statistical Manual of Mental Disorders, Fourth Edition* (DSM–IV) (American Psychiatric Association [APA] 1994).

Alcohol abuse generally refers to a maladaptive drinking pattern involving recurrent difficulties in one or more of the following areas: failure to fulfill major obligations (e.g., the demands of school, employment, or parenthood), alcohol use in physically hazardous situations (e.g., driving, boating, or skiing), legal difficulties caused by drinking (e.g., arrest for driving while intoxicated), and alcohol-related social consequences (e.g., engaging in physical fights) (APA 1994).

Criteria for alcohol dependence include tolerance, withdrawal,^2^ impaired control over alcohol use (e.g., difficulty cutting down or drinking more than intended), narrowing of nondrinking activities (i.e., alcohol plays a pervasive role in many life activities), and continued use of alcohol despite knowledge of associated adverse consequences (APA 1994). A diagnosis of alcohol dependence (i.e., alcoholism) precludes the diagnosis of alcohol abuse.

One-year prevalence for alcohol abuse is the more common of the two alcohol-related disorders (table 2) (Grant 1994). In total, just under 9 percent of the adult (i.e., age 18 and older) U.S. population meet 1-year prevalence criteria (according to DSM–IV criteria) for either alcohol abuse or dependence (Grant 1994). One-year prevalence rates for alcohol abuse and dependence among adults in the United States are higher for men than for women. Rates of alcohol abuse and dependence are disproportionately higher among people between the ages of 18 and 29 compared with other age groups. Almost one-fourth of young men in the United States meet the criteria for 1-year prevalence for an alcohol-use disorder. Just over one-half of these men meet the diagnostic criteria only for alcohol abuse, and the remaining men meet the criteria for alcohol dependence as well. Prevalence rates for alcohol-use disorders are considerably lower for women in this age range. These rates are higher among white men and women compared with their nonwhite peers (Grant 1994), consistent with ethnic group differences in prevalence of consumption and binge drinking.

### Drinking and Driving

Young adults are overrepresented among alcohol-related traffic fatalities (National Highway Traffic Safety Administration 1994). Although young-adult drivers ages 16 through 25 make up only 15 percent of U.S. licensed drivers, they constitute 28 percent of drinking-driver fatalities (Campbell et al. 1995). Furthermore, youth between the ages of 16 and 24 make up 30 percent of all alcohol-related driving fatalities, including fatalities of drivers, passengers, and nonoccupants (Campbell et al. 1995). Inexperience with both driving and drinking may contribute to this disproportionate rate.

Young-adult drivers (ages 16 to 24) involved in automobile accidents are more likely to have a lower blood alcohol concentration (BAC) than older drivers (Campbell et al. 1995). This pattern may reflect greater impairment and lower alcohol tolerance among less experienced drinkers who drive after drinking.

### Other Alcohol-Related Consequences

Many studies of young adults’ alcohol-consumption patterns have used samples of college students, limiting researchers’ ability to generalize study findings to other young-adult populations. However, because the most detailed information about the nature and prevalence of alcohol-related consequences within this age group has been gathered from college students, some examples of the more comprehensive studies are presented here.

Hanson and Engs (1992) administered four surveys over a 10-year period to students from 4-year colleges in the United States. In the most recent of these surveys, 16.9 percent of the respondents who drank indicated that within the past year, they had become involved in a fight after consuming alcohol, and 10.3 percent indicated that they had engaged in ill-advised behavior, such as damaging property or sending false fire alarms, after drinking. The 1-year prevalence of each of these consequences increased significantly over four assessments from 1982 to 1991 (Hanson and Engs 1992). Sociocultural and other factors may affect the generalizability of these findings to today’s college students. The findings are useful, however, as an approximation of general trends in the nature and prevalence of alcohol-related consequences that college students experience.

Wechsler and colleagues (1994, 1995*a*,*b*) conducted a national survey of college students to determine the extent of health-related and behavioral consequences associated with binge drinking. The minimum binge-drinking criteria in this study were five drinks per occasion for men and four for women. Consequences reported more often by binge drinkers included arguments with friends, getting hurt or injured, and damaging property. The frequency of such consequences was related to the frequency of the subject’s binge drinking. Forty-seven percent of the students who frequently engaged in binge drinking (three to four binges in the past 2 weeks) reported five or more alcohol-related consequences during the school year, compared with 14 percent of those who were less frequent binge drinkers and 3 percent of drinkers who did not binge within the past year. Among these consequences were personal injury, property damage, unplanned sexual activity, and unprotected sex.

Although specific consequences of drinking have not been reported for noncollege young-adult samples, it is likely that many of the same consequences would occur given comparable levels of consumption.

## Binge drinking and the Maturation Process

Although the pattern of drinking and the amount consumed remain similar from late adolescence through young adulthood, most heavy-drinking young adults appear to “mature out” of abusive drinking patterns as they pass into subsequent stages of adulthood.

Schulenberg and colleagues (1996) evaluated changing patterns of binge drinking among young adults who were initially assessed during their senior year in high school and who completed three subsequent biennial assessments. Frequent binge drinking was defined as two or more five-drink binge episodes in the past 2 weeks. Statistical analysis of the results confirmed the existence of six distinct patterns of change^3^ in frequent binge drinking over time. These differing patterns correspond to classifications of problem drinking as well as to patterns of maturation. Frequent binge drinkers who maintained their heavy drinking levels throughout the survey period made up 6.7 percent of this sample. The researchers concluded that this group corresponded most closely to the antisocial type of alcoholism described by Zucker (1994), in which problem drinking is only one of a constellation of behavioral problems that begin in childhood and continue through adulthood.

Another group of respondents who were initially frequent binge drinkers were no longer frequent binge drinkers at the time of the final assessment (Schulenberg et al. 1996). This pattern of decreasing frequency may correspond to what has been called the “developmentally limited” type of problem drinking. This type resembles antisocial drinking in most respects except for its limited duration. Zucker (1994) relates this type of alcohol problem to normal adolescent development and the process of individuating from parents.

Most young adults in the study who engaged in binge drinking matured out of this pattern of drinking over time. Some of these respondents decreased their binge drinking gradually, whereas others experienced a brief (“fling”) period of binge drinking between periods of no binge drinking. Some young adults increased the frequency of drinking over time, perhaps responding to the increased opportunities for drinking available to young adults; these subjects may or may not mature out of this pattern over time. Understanding the changes in drinking patterns as these individuals approach mid-adulthood may clarify the processes involved in later onset binge drinking.

Marital status, ethnicity, and number of years of education may be predictive of continued binge drinking. Those who had not married by age 23 or 24 were generally more likely to continue or increase episodes of binge drinking. Caucasians tended to be the ethnic group most likely to maintain or increase binge drinking. Men who had more years of education or whose parents had more years of education were more likely to continue or increase their binge-drinking frequency. Men who engaged in frequent reckless driving were less likely to mature out than nonreckless drivers (Schulenberg et al. 1996), a finding that is consistent with the antisocial types of alcohol problems. Women who had received financial support from their parents were more likely to increase risky patterns of drinking than women receiving no support (Schulenberg et al. 1996).

Changes in marital and employment status also have been associated with changes in consumption. Specifically, Temple and colleagues (1991) found an association between becoming married and decreases in typical quantity of consumption across age groups and for both men and women. Remaining or becoming single, on the other hand, was associated with increased consumption for men and women under age 40 (Temple et al. 1991).

Regarding employment status, Temple and colleagues (1991) found a modest association between the initiation of employment and increased consumption for young men and women, possibly because of increased financial resources. This finding appears to be contrary to theories which propose that increases in adult responsibilities, such as employment, would decrease consumption levels. It is unclear, however, whether changes in the pattern of drinking among newly employed young adults occur in response to employment (e.g., typical quantity may increase, but weeknight frequency of consumption may decrease). Becoming unemployed was associated with increased typical quantity of consumption for men under 40 but not for young women. Chronic or long-term unemployment was associated with decreases in consumption for young-adult women (under age 40) but was associated with an increase in typical quantity of consumption for young men (Temple et al. 1991).

## Risk Factors for Alcohol-Related Problems

Given that the highest prevalence of heavy drinking and associated problems occurs during the late teens and early to mid-twenties, young adulthood may be considered a risk period for heavy drinking and related consequences. Demographic risk factors were discussed earlier; other risk factors are discussed below.

### Behavioral and Psychological Factors

Of the psychological risk factors, personality characteristics, such as impulsivity and lack of behavioral control, also have been implicated as risk factors for heavy alcohol use and attendant negative consequences. A history of childhood behavior problems has been associated with greater increases in alcohol use and symptoms of alcoholism among youth entering college (Baer et al. 1995). Some young adults with histories of behavior problems may develop temporary problems with alcohol as they pass through the risk periods of adolescence and young adulthood; others may go on to develop the more chronic disorder of alcohol dependence.

Beliefs about the positive or negative effects of alcohol consumption are known as expectancies. Expectancies relate not only to alcohol’s mood-altering effects but also to broader issues of social behavior and acceptance by one’s peers. Young adults who expect alcohol to have a positive effect have been shown to have heavier patterns of consumption compared with those without such expectancies (Mooney et al. 1987); they therefore may be more likely to abuse alcohol. Positive expectancies for alcohol effects have been shown to increase with age during adolescence (Christiansen et al. 1985). Furthermore, adolescents ages 12 through 19 in treatment for alcohol abuse have been shown to have higher positive outcome expectancies than their nonabusing peers (Brown et al. 1987).

### Social and Environmental Factors

Social risk factors may include norms regarding use and acceptability of alcohol, social and residential environment, and family influences. Residence in a fraternity or sorority and adoption of a party-centered lifestyle are strong predictors of college binge drinking (Wechsler 1995*b*). In addition, college students have been shown to overestimate drinking norms (Baer et al. 1991), a perception that may promote heavier or abusive levels of consumption. Abusive drinking also may be facilitated when young adults emulate the drinking habits of heavy-drinking peers (Collins et al. 1985).

Examples of environmental factors that may affect consumption rates include minimum legal drinking age (MLDA) and the cost of alcohol. Davis and Reynolds (1990) evaluated the impact of legislation raising the MLDA in New York State from 19 to 21. Analysis of alcohol consumption among a sample of university students showed a slight moderation of drinking levels overall, especially among the heaviest drinkers. However, students reported increased negative consequences of drinking, such as physical injury. The greater number of injuries may be caused by increased drinking in less controlled environments, such as private rooms and un-monitored parties (Davis and Reynolds 1990).

Computer simulation is a research tool that has been used to predict trends in alcohol consumption based on cost. Studies using computer simulation have concluded that raising the cost of alcohol through taxation would significantly decrease binge and frequent drinking among youth (Grossman et al. 1994). These results implicate the low cost of alcohol as a risk factor for abusive drinking among youth.

### Biological Factors

A family history of alcoholism has been evaluated as a risk factor for alcohol problems in young adults. Epidemiological studies show that the offspring of alcoholics are three to four times more likely to develop alcohol problems than are the offspring of nonalcoholics, regardless of the environment in which they are raised (Goodwin 1988). Consequently, the children of parents with alcohol disorders may be at increased risk for developing alcohol problems in adulthood through a genetic predisposition (Schuckit and Smith 1996; Sher 1991).

Family history as a risk factor for alcohol problems may be mediated, in part, by a genetically influenced decreased sensitivity to alcohol (Schuckit and Smith 1996). Independent of consumption level during young adulthood (i.e., in the early twenties), men who had extremely low responses to alcohol (measured as lower levels of self-reported intoxication and as changes in certain physiological functions) were more likely to meet diagnostic criteria for alcohol abuse or alcohol dependence by age 31 compared with men who had higher levels of response. Family history and low level of alcohol response independently contributed to later problems with alcohol (Schuckit and Smith 1996).

However, a few well-designed studies examining genetic predisposition to alcohol problems among young adults (and college students in particular) have not supported a genetic-vulnerability hypothesis (Alterman et al. 1986, 1990; Schuckit and Sweeney 1987). These studies failed to find differences between the consumption level and number of alcohol-related problems between sons of problem drinkers (i.e., high-risk subjects) and low-risk control subjects in this age group (Alterman et al. 1986). One exception is Schuckit and Sweeney (1987), who found that those at high risk (as indicated by family history) reported relatively more alcohol-related problems at a level of consumption comparable to control subjects. Family history has not been correlated with consumption patterns, alcohol-related consequences, or symptoms of alcoholism in college students at risk for alcohol abuse (Baer et al. 1995).

Individual differences in responsiveness to alcohol’s stress-reducing properties have been posited as a risk factor for alcohol abuse. Subjects in whom alcohol greatly diminishes certain physiological responses associated with stress may be more prone to alcohol abuse than those who exhibit weaker responses (Sher 1987).

## Protective Factors and Intervention

In general, the presence of multiple risk factors may be considered to be additive in evaluating risk status. For example, with all other things equal, a 20-year-old Caucasian man who frequently drank five or more drinks in high school would be viewed as having a higher risk for alcohol abuse or related consequences during young adulthood than a 20-year-old Caucasian man who did not drink excessively in high school. However, changes in roles may serve as “protective factors” leading to discontinuation of heavy use or maintenance of lower risk drinking practices. The addition of adult roles (e.g., spouse, parent, or employee) may be incompatible with and preclude the continuation of a heavy pattern of consumption.

Programs designed to reduce risks and consequences associated with young adults’ drinking have been effective in reducing consumption and consequences. Baer (1993) documented significant reductions in drinking among heavy-drinking (i.e., high-risk) college freshmen following single 1-hour motivational interviews. In these sessions, each student met individually with a staff person who provided concrete information about the student’s drinking patterns, expectancies, and risks as well as suggestions for risk reduction. Followup assessments 1 and 2 years later found that high-risk subjects who had participated in interview sessions were drinking in a less risky manner compared with a group of high-risk subjects who had not participated in such interviews. Participants also reported fewer alcohol-related consequences, such as academic impairment and interpersonal difficulties (Marlatt et al. 1995).

The Lifestyle project (Baer 1993; Marlatt et al. 1995) and the Alcohol Skills Training Program (Kivlahan et al. 1990), both of which were developed at the University of Washington, use knowledge of risk factors (e.g., peer influences, expectancies, and social environment) to facilitate motivation for alcohol-risk reduction or earlier maturing-out among high-risk subjects. What remains to be seen is whether such interventions are effective with young adults in other environments, such as primary health care, residential, and military settings, as well as among youth remanded to alcohol treatment by the courts.

## Figures and Tables

**Table 1:** Prevalence (%) of Alcohol Use and Heavy/Binge Drinking Among Young Adults

Study	Age Group	Alcohol Use (%)			

		Lifetime	Annual	Past 30 Days	Heavy/Binge Drinking^1^
Monitoring the Future^2^	19–28	91.2	83.7	67.7	33.7
National Household Survey on Drug Abuse	35+	87.6	64.6	48.8	4.2
	26–34	92.4	81.0	62.8	7.3
	18–25	87.1	79.0	59.3	10.4
	12–17	41.2	35.2	18.0	1.3

1 Binge drinking was defined in the Monitoring the Future study as consumption of five or more drinks at least once during the 2-week period preceding the survey. Heavy drinking in the National Household Survey on Drug Abuse was defined as consumption of five or more drinks on each of 5 or more days in the past 30 days.

2 Survey of high school graduates.

SOURCE: Adapted from Johnston et al. 1996 and Substance Abuse and Mental Health Services Administration 1994.

**Table 2:** Prevalence (%) of Alcohol-Use Disorders^1^ in the General Population and Among Young Men and Women Ages 18 to 29

Alcohol-Use Disorder	General Population	Men		Women	
		
	18+	18+	18–29	18+	18–29
Abuse	4.72	7.49	12.70	2.22	8.85
Dependence	3.84	5.78	11.01	2.09	4.98
Total Alcohol-Use Disorders	8.56	13.27	23.71	4.31	13.83

1 Criteria for alcohol abuse and dependence as outlined in the *Diagnostic and Statistical Manual of Mental Disorders, Fourth Edition* (American Psychiatric Association 1994).

SOURCE: Adapted from Grant 1994.

## Footnotes

1 Prevalence is the number of cases of a disease existing in a given population within a given period of time (e.g., the week or month preceding the interview). Lifetime prevalence refers to occurrence of the disease at any time in a subject’s life.

2 Tolerance indicates that increasing quantities of a drug are required to produce a given effect. Withdrawal occurs upon discontinuation of heavy drug use and may be characterized by anxiety, sweating, elevated blood pressure, hallucinations, and, in severe cases, seizures.

3 The pattern of “never binge drinking” will not be discussed here.
